# Translating mentoring interventions research into practice: Evaluation of an evidence-based workshop for research mentors on developing trainees’ scientific communication skills

**DOI:** 10.1371/journal.pone.0262418

**Published:** 2022-02-02

**Authors:** Erin K. Dahlstrom, Christine Bell, Shine Chang, Hwa Young Lee, Cheryl B. Anderson, Annie Pham, Christine Maidl Pribbenow, Carrie A. Cameron

**Affiliations:** 1 Department of Behavioral Science, The University of Texas MD Anderson Cancer Center, Houston, TX, United States of America; 2 Wisconsin Center for Education Research, University of Wisconsin-Madison, Madison, WI, United States of America; 3 Division of Cancer Prevention and Population Sciences, Cancer Prevention Research Training Program, The University of Texas MD Anderson Cancer Center, Houston, TX, United States of America; 4 Department of Epidemiology, The University of Texas MD Anderson Cancer Center, Houston, TX, United States of America; 5 Division of Social Science, Behavioral Research Laboratory, Rice University, Houston, TX, United States of America; Centers for Disease Control and Prevention, UNITED STATES

## Abstract

A key part of keeping doctoral and postdoctoral trainees in STEM research careers is mentoring. Our previous research indicates that mentoring trainees in scientific communication (SC) skill development increases research career intention through two social-cognitive constructs, self-efficacy in and outcome expectations for acquiring SC skills, as well as science identity. While many mentor training interventions exist, no programs focus on developing SC skills specifically. The “Scientific Communication Advances Research Excellence” (SCOARE) program trains mentors to address trainee scientific communication (SC) skill development as an innovative approach to increase trainee research career persistence. The SCOARE training is a half-day workshop for faculty mentors of research trainees at five sites nationally. Informed by previous research, workshop content focuses on practical, effective mentoring strategies to develop trainee speaking and writing skills. Anonymous evaluation data collected after each workshop indicates participant satisfaction and reported positive increases in skills and knowledge in applying new and various techniques when mentoring trainees (skills) and how linguistic bias influences our perception of others (knowledge). This article outlines the research-based development of the SCOARE program, the first two years’ of workshop evaluations showing positive increases in skills and knowledge, and lessons learned to increase participant satisfaction with the program.

## Introduction

In spite of over a decade of national efforts to mitigate attrition of doctoral and postdoctoral trainees from STEM research careers, at least 25% of STEM doctoral students (and a disproportionately larger percentage of underrepresented groups) move away from an academic research career during their doctoral studies [1]. Research has shown that a key part of the solution is mentoring. Interventions to enhance mentoring quality have been shown to influence factors related to trainee research career intention and persistence such as self-efficacy, outcome expectations, and science identity [2–7]. One important facet of mentoring that has been shown to have a measurable impact on these factors and on intention to remain in a research career overall is scientific communication (SC) skill development [8–10]. STEM faculty rarely have training in the dynamics of communication skill development, however, and most persevere in their mentoring unaware of the wealth of methods developed in communication-related fields. Here, we describe the development of a novel intervention designed to support mentors in understanding the role of SC in trainee career development and the evaluation results from its first two years of implementation. The intervention teaches linguistically-motivated, accessible techniques for approaching trainee SC skill development strategically and efficiently.

The intervention’s design is based on previous research that adapted Social Cognitive Career Theory [SCCT; 11, 12] to investigate the relationships between SC skills, mentoring, and career intention, informed by sociolinguistic theories of identity development [13–16]. SCCT posits that an individual’s self-efficacy, or sense of competence in a particular domain, and outcome expectations, the anticipated consequences of these behaviors, foster interest and goals for pursuing a career in a particular domain. We define SC as writing, speaking, and presenting, with speaking defined as non-scripted communication (asking questions, speaking up) and presenting as scripted (formal research talk). It has previously been shown that not only are SC skills foundational, practical skills that trainees will use throughout their future careers, they are also psychologically linked to science identity [10]. Two previous studies, which used cross-sectional surveys and longitudinal surveys of research trainees, indicated that mentoring trainees specifically in the development of SC skills, as well as active engagement of trainees in all modes of SC, increase trainees’ intention to remain in research careers [8, 9]. The social-cognitive constructs of self-efficacy in and outcome expectations for acquiring SC skills, together with science identity, predicted this increase in research career intention [10].

While a variety of mentor training interventions have been created with the goal of strengthening the participation in and persistence towards research careers of a diverse group of trainees, no programs had been developed that focused specifically on mentoring in SC skills and their impact on trainee research career intention. A workshop intervention providing faculty with tools to mentor their trainees in SC skills was the next step. With support from the National Institute of General Medical Sciences, we created the ‘Scientific Communication Advances Research Excellence’ (SCOARE, R25-GM125640) program as the translation of the research into practice. This report outlines the development of the SCOARE program curriculum and its evaluation, the delivery and implementation of the workshops, results from the first two years of workshop participant evaluations, and the adaptation of the curriculum based on evaluation results.

## Program design and implementation

The SCOARE program was originally developed for an audience of biomedical research faculty who directly mentor doctoral and postdoctoral trainees. It has 4 components: (1) a half-day workshop for research faculty and staff mentors focused on how to mentor trainee SC skill development; (2) an external evaluation of the workshop and its impact (conducted immediately after workshop attendance); (3) an associated research study for participating mentors and up to five of their doctoral- or postdoctoral-level trainees to assess the effectiveness of the workshop for mentors (conducted before and six months after workshop attendance; approved by The University of Texas MD Anderson Cancer Center IRB, Protocol 2018–0206); and (4) a SCOARE facilitator training workshop, or “Train-the-Trainer.” This report addresses workshop development and evaluation data only (Components 1 & 2). Research study data (Component 3) is in preparation, and the associated Train-the-Trainer (Component 4) is currently being executed.

### Creation of workshop learning objectives

**Fig 1:**
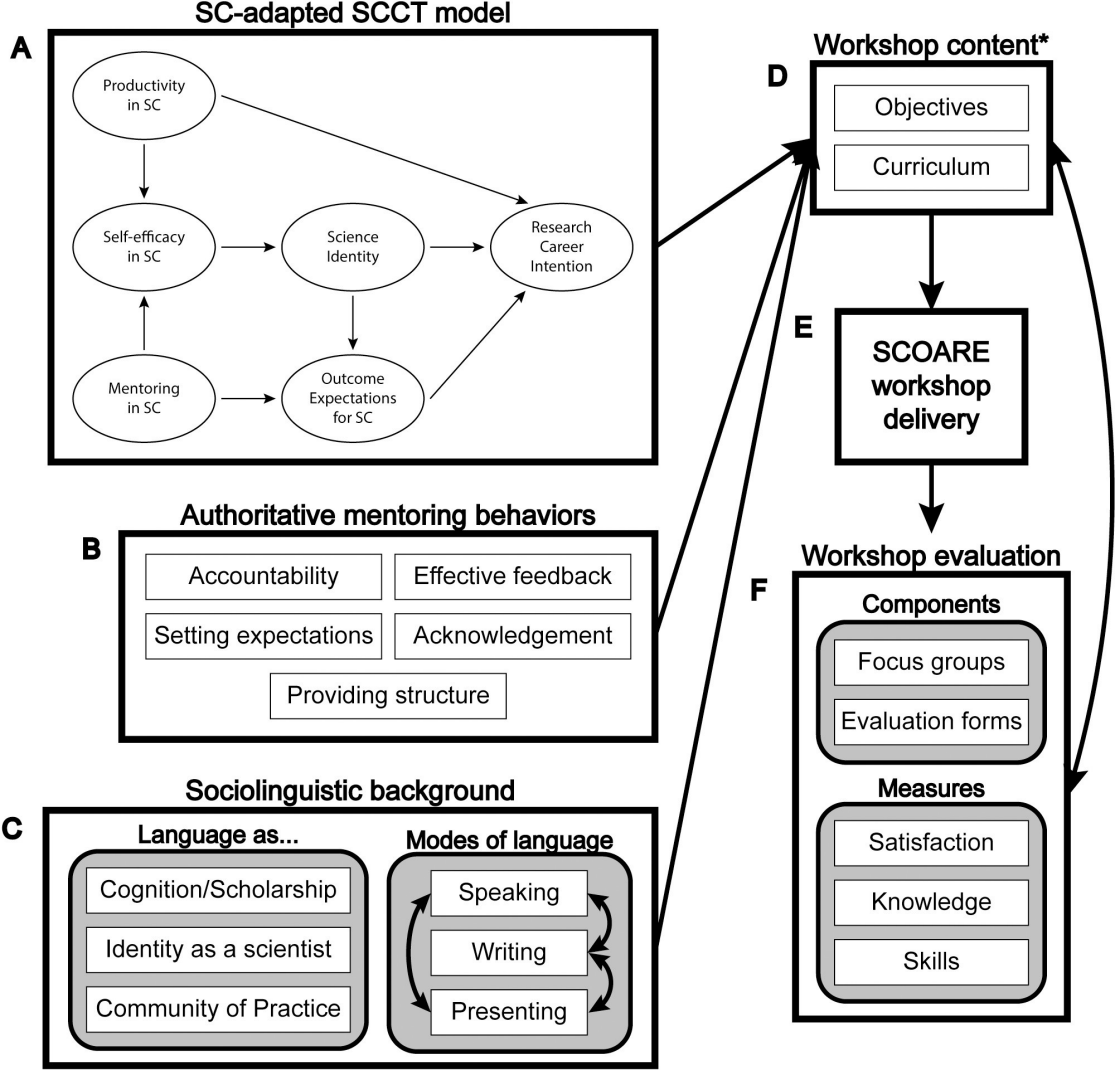
Flowchart of SCOARE program design and evaluation. The previously developed SC-adapted SCCT model (A), defined authoritative mentoring behaviors encompassed in the model (B), and sociolinguistic background including modes of language use and the social, psychological, and affective aspects of communication (C) were used to develop the workshop content, including objectives and curriculum (D). *Note, full workshop content, including units and the topics therein can be found in Table 1. Workshop content was delivered via the SCOARE workshop (E), then evaluated (F), and the evaluation findings were used to adapt and fine-tune the workshop content.

As noted above, the SCOARE workshop was designed based on the results of over a decade of research on mentors and trainees which culminated in an adapted SCCT model [(Fig 1A); 10]. In this model, “Productivity in SC” is one of two independent variables and refers to the degree of general participation in writing, speaking, and presenting a trainee engages in, *regardless of peer review or publication*. In other words, this construct represents how often trainees speak up, engage in any level of scholarly writing, or present to a group, such as at a national conference. “Mentoring in SC” is the second independent variable and encompasses a variety of behaviors that trainees reported they experienced with their mentors regarding SC. Together, productivity in SC and mentoring in SC influence “Self-efficacy in SC,” or a trainee’s perception that they are competent at SC, which influences “Science Identity,” or the sense that one has of ‘belonging’ in a science career. Productivity in SC also directly predicts research career intention. Finally, science identity and mentoring in SC predict “Outcome Expectations for SC,” or trainees’ anticipated consequences of engaging in SC, which then predicts career intention. See Cameron, Lee [10] for further detail.

The model’s key social-cognitive constructs and their relationships informed the creation of workshop learning objectives for attendees. More specifically, the workshop objectives focus on training mentors to impact their trainees through the two independent variables in the model—productivity in SC and mentoring in SC. The objectives are:

1. To understand their role and perspective as a mentor versus that of a trainee in SC development;
2. To learn how to set expectations, create structure, and create and apply strategies to increase trainee engagement in SC development;
3. To learn to deliver useful and appropriate feedback; and
4. To create, adapt, and personalize mentoring strategies to use with their own trainees.

Objectives 2 through 4 focus on developing mentoring behaviors to impact trainees. Research results indicated that trainees whose mentors engaged in more mentoring behaviors had higher self-efficacy and outcome expectations in SC, both of which contribute to a higher research career intention. The mentoring behaviors encompassed in the model are characterized as ‘authoritative’ [(Fig 1B); 17]. This characterization was adapted from research into effective parenting styles [18, 19] and is comprised of both ‘demanding’ behaviors, i.e., having and communicating high expectations of trainees, and ‘responsive’ behaviors, i.e., providing feedback, support, and encouragement.

### Workshop curriculum design and overview

At the start of the workshop, participants receive an overview of how the research described above informed the workshop curriculum development, including an overview of the adapted SCCT model (Fig 1A). Inclusion of the scientific underpinnings and research basis was purposeful, to engage faculty mentors who themselves are researchers. The model is referenced throughout the workshop to emphasize the pathways through which mentoring in SC could influence trainee research career intention.

In addition to inclusion of its scientific underpinnings, the workshop curriculum contains three additional key pieces of novel content. The first is a short primer on the role of language in identity development and entry into a Community of Practice [20, 21]. This is based on sociolinguistic background within the context of science identity and the community of scientists. The second is a unit on the dynamics of language use in social interaction and within the research environment and on the process of how we learn communication skills. This unit addresses three separate modes of SC—writing, speaking, and presenting—including how each is distinct from one another and the development of all three. It provides insights on how to approach SC mentoring through the critically important social, psychological, and affective aspects of communication, which are widely overlooked and afford easily accessible opportunities for positive intervention (Fig 1C). The last, and largest, is a unit on developing concrete, ‘authoritative’ mentoring strategies, as described above, to use in trainee SC skill development. Subsections of this unit and a more detailed description of workshop units and content can be found in Table 1.

**Table 1:** SCOARE workshop units and content.

Unit	Content & Activities
Workshop Introduction	Content:
	• Facilitator and team introductions
	• Workshop logistics, including walkthrough of workshop participant binder and resource website
	• Language and unconscious bias
	• The role of language in identity, cognition, and communication
	Activities:
	• Participant introductions (full group)
	• Biggest SC challenges (table discussion)
Research Background	Content:
	• Summary of our 10 years of mentoring research
	• Adapted SCCT model
	• Data on trainee-perceived judgment on language variety use in the research environment
	Activities:
	• Common misconceptions about SC (review of pre-workshop voting activity, group discussion)
	• Case Study: Mentoring Scott (table and group discussion)
Language Use	Content:
	• Non-native English speakers
	• Scientific style
	• Three modes of SC
	Activities:
	• Freewriting (individual)
	• Post-it feedback (individual)
Authoritative Mentoring	Content:
	• Setting expectations and creating structure
	• Accountability
	• Acknowledgement
	• Giving effective feedback
	• Scaffolding SC learning
	• Increasing productivity and engagement in SC
	Activities:
	• Your experiences with authoritative or non-authoritative mentoring (table discussion)
	• Exercise on organizing trainee thoughts for a research report (table and group discussion)
	• Engagement strategies (table discussion)
Moving Forward	Activities:
	• Finalizing mentoring plan (individual and pairs)
	• Lingering questions (full group)

The workshop was designed and adapted with the help of an instructional designer to balance time spent on didactic workshop sections, discussion, and activities. Upon arrival but before the workshop begins, participants vote on the truth or falsity of three common, widely held beliefs about SC (which are then revisited in the research unit). Throughout each of the workshop units, participants are engaged through (1) discussions of varying group sizes, ranging from pairs to the full group; (2) a case study examining language variety and its implications in the research environment; (3) an extended exercise on how to help a trainee organize ideas for a research report, introduced in Year 2 to address overarching mentors’ concerns about trainee writing and the mentors’ need to re-write trainee manuscripts; and (4) a ‘Brainstorming Sheet’ they complete throughout the workshop with mentoring strategies that resonate with them. As a final activity, participants use their Brainstorming Sheet to formulate a mentoring plan for moving forward and discuss it with another participant. We also include flipchart activities at the start and middle of the workshop to get a measure of the audience before exposure to the SCOARE workshop material and partway through, and to manage audience expectations.

Comprehensive binders are provided to participants to take home with all activities and materials from the workshop, including copies of the slides, handouts that accompany the activities described above, additional resources for mentors, and copies of relevant scholarly work. Participants are also given free copies of two books to use as resources: *Engaging Ideas*: *The Professor’s Guide to Integrating Writing*, *Thinking*, *and Active Learning in the Classroom* by John Bean and *TED Talks*: *The Official TED Guide to Public Speaking* by Chris Anderson.

After the workshop, we provide online follow-up support to ensure the integration of the learning into mentors’ daily activities via a resource website. We initially used Study, an MD Anderson-licensed version of the digital learning management platform Canvas, but after noting low engagement have moved the resources to a Squarespace website to be more accessible to participants outside of MD Anderson. The Squarespace site is not password protected to allow easy access to all past participants but is set to an unlisted URL until the conclusion of our research study to prevent future study participants from engaging with the materials before attending the workshop. It provides well-organized and easy-to-locate resources that align with the workshop content, including relevant scholarly articles, useful handouts and websites for trainees, and instructional content for mentors on topics such as setting up writing retreats. Participants are also given a handout that correlates the resources on the website to the material covered in the workshop.

## Methods

### Recruitment, workshop delivery, and research study

In the first two years, 11 half-day workshops were delivered at five sites nationally: three at The University of Texas MD Anderson Cancer Center (Houston, TX), two at Georgia State University (Atlanta, GA), two at the University of Colorado Boulder (Boulder, CO), one at Northwestern University (Chicago, IL), one at the Big Ten Academic Alliance (Chicago, IL), one at the Gulf Coast Consortia (Houston, TX), and one at the University of Wisconsin-Madison (Madison, WI). Workshops were initially three and a half hours long at the start of Year 1 to accommodate the busy schedules of faculty mentors but were extended to a final length of five hours by the end of Year 2. Local site partners at each institution organized workshop logistics, including room booking and catering, and coordinated recruitment with materials provided by the SCOARE team. To recruit workshop participants, site partners sent workshop flyers, a workshop description, and registration information to relevant listservs of faculty mentors and research networks at their institution and surrounding institutions. While recruitment was targeted at biomedical research faculty and staff who work with graduate and postdoctoral-level trainees, the workshops were open to all STEM faculty and staff, and participants ranged from faculty at medical institutions to administrators who work with undergraduates. Starting in Year 2, previous workshop attendees were also invited to forward information about the current year’s workshops to their own networks.

Workshops were limited to 20 participants, seated at small tables of 3–6, to allow for an adequate level of interaction from all participants during group activities and discussions. While workshop attendance was open to all STEM faculty and staff, only research faculty mentors who were directly mentoring at least one doctoral- or postdoctoral-level research trainee were eligible to participate in the associated research study. Attendees participating in the study were given priority for workshop spaces, but research study participation was not required for workshop attendance. Of the 260 staff and faculty mentors who initially started registration to attend a workshop in Years 1 and 2, 245 completed the registration, and 167 attended a workshop. Notably, from Year 1 to Year 2 there was a 13% increase in completed workshop registrations, including a 20% increase in those registered for both the workshop and research study, and a 26% increase in workshop attendance. Participant disciplines ran the gamut from reproductive endocrinology to computer science, however the majority of participants work in basic science research, with significant proportions of participants in biomedical/bioengineering and clinical disciplines. See Table 2 for additional workshop participant demographics.

**Table 2:** Workshop participant demographics, Years 1 & 2 (N = 167).

	Category	Frequency	Percentage
Gender	Female	101	60
	Male	61	37
	Other/Unknown	5	3
Ethnicity	Hispanic/Latino	19	11
	Non-Hispanic/Latino	143	86
	Unknown	5	3
Race	Asian or Asian American	23	14
	Black or African American	9	5
	White or European American	118	71
	Other/ More than one race/ Unknown	17	10
Academic rank	Assistant Professor	59	35
	Associate Professor	41	25
	Professor	39	23
	Other	19	11
	Unknown	9	5
Years of mentoring experience	0–5 years	55	33
	6–10 years	40	24
	11–15 years	26	16
	16–20 years	18	11
	21+years	20	12
	Unknown	8	5
Number of trainees currently mentored	0–4	71	43
	5–8	50	30
	9–14	17	10
	15+	18	11
	Unknown	11	7
Workshop location	Atlanta	35	21
	Boulder	24	14
	Madison	19	11
	Chicago	23	14
	Houston	66	40
Study participation	Yes	109	65
	No	58	35

### Workshop evaluation design

External evaluation was designed and conducted by The Learning through Evaluation, Adaptation, and Dissemination (LEAD) Center at the Wisconsin Center for Education Research at the University of Wisconsin-Madison. Evaluators from the LEAD Center were engaged from the beginning of workshop curriculum design and developed the initial evaluation design, focus group plan, and survey instrument. After the start of workshop delivery, evaluators carried out the evaluation plan and data analysis.

The mixed-methods evaluation plan entailed observation of workshops, post-workshop focus groups in Year 1 only (data were used for formative evaluation and are not reported in this article), and post-workshop surveys for participants (Fig 1F). The evaluation plan was designed to answer four evaluation questions: (1) How satisfied are workshop participants; (2) Does the developed SCOARE curriculum and workshop meet its learning objectives; (3) What are participant gains in knowledge and skills from the workshop; and (4) Will participants implement what they have learned in the workshop. The purpose was to review the evaluation findings after each workshop to ensure participants felt the workshop learning objectives were being met and to adapt the workshop to see improvements in participant ratings of satisfaction, knowledge, skills, and self-efficacy in the next workshop and observe overall trends. Informed consent was not part of the evaluation process because the University of Wisconsin-Madison Institutional Review Board does not consider evaluation as falling under the definition of research and notes that “program evaluation and QI [quality improvement] projects can be published or presented as such, but they cannot be described as research studies” [22]. Steps taken to ensure ethical publication included the anonymous collection of evaluation data and publishing findings in the aggregate without direct participant quotes.

The evaluator(s) observed four of the five workshops in Year 1 and two of the six workshops in Year 2. The purpose of observation was for the evaluators to gain firsthand experience of the workshop and witness participant engagement.

All participants, regardless of participation in the associated research study, completed paper surveys at the end of the workshop, then placed their completed evaluations in a folder out of view from the facilitators. Paper surveys were scanned and electronically sent to the evaluator when not in attendance. The evaluator used Microsoft Excel to analyze and Microsoft Word to report the data to the facilitators as soon as possible in order to make adjustments before the next workshop. At the end of each year, evaluation data from each site were combined, compared, and examined by the team and site partners.

The post-workshop evaluation survey (S1 File), which is separate and distinct from the research study surveys, was tailored to the content and terminology of the SCOARE program. It evaluated three levels of training results: (1) level of satisfaction, or reaction to the training; (2) level of learning; and (3) level of behavior, or intention to implement [23–25]. The form included questions about the meeting of workshop objectives, self-rated attendee skills in and knowledge of workshop content retrospectively before and after the workshop, likelihood of using mentoring strategies after the workshop, and self-rated attendee capability to implement strategies learned in the workshop.

The post-workshop survey used multiple choice or Likert-type scale, open-ended, and retrospective pre-post test item design. Retrospective methodology was used because it is appropriate for adult audiences and short interventions, and its goal is a short-term, quick reflection on personal growth related to the program, not a comprehensive measure of participant learning [26]. Rather than waiting for the results of the research study, this gave facilitators an immediate snapshot of how participants think they are doing, which was useful for rapid improvement in the first two years of the program.

The use of retrospective methodology is sound for short-term interventions with adults. It can reduce response-shift bias and recall bias [26], which is advantageous for short interventions [27]. It was also not feasible to administer a pre-workshop evaluation survey to participants that would include specialized terminology introduced in the workshop and require an assumption that participants are cognizant of what they do not know and their personal misconceptions about mentoring styles, skills, and knowledge [26, 28, 29]. Wilcoxon Signed Rank tests were conducted with IBM SPSS statistics 24 (IBM Corp, Armonk, NY) to compare the means of the retrospective pre- and post-workshop responses.

The evaluators made minor revisions to the survey instrument after Year 1 and again between Year 1 and Year 2. The revisions reflected adaptations made to the workshop: re-articulated learning objectives, and retrospective items at a more specific level of understanding to assess if participants were gaining skills and knowledge in both scientific writing and scientific speaking/presenting separately. Changes helped add focus to strategies for scientific writing, the topic of greatest interest to participants and for which they expressed the most need. Throughout the workshop participants heard a repeated theme that speaking and presenting are distinct from each other and equally important to scientific writing.

## Results

Of the 74 mentors who attended the workshop in the first year, 67 (91%) completed the post-workshop evaluation. Of the 94 mentors who attended the workshop in the second year, 93 (99%) completed the post-workshop evaluation. Of the evaluations, 40 (60%) of the first year’s and 62 (67%) of the second year’s came from research study participants. Evaluation survey data can be found in the S1 Dataset.

### Evaluation question 1: How satisfied are workshop participants?

Overall, participant agreement with multiple areas of satisfaction increased from Year 1 to Year 2. Ninety-two percent (n = 62) of the participants from Year 1 agreed or strongly agreed that they learned a lot from the workshop. In Year 2, 99% (n = 92) of participants agreed or strongly agreed they learned a lot from the workshop. In Year 1, 97% (n = 64) of participants agreed or strongly agreed the workshop increased their understanding of mentoring trainees in SC; in Year 2, 100% (n = 93) of participants agreed or strongly agreed. Eighty six percent (n = 58) of Year 1 participants agreed or strongly agreed they would recommend this workshop to a colleague, which increased to 96% (n = 90) saying they would recommend it in Year 2. Eighty nine percent (n = 60) of Year 1 participants agreed or strongly agreed they were satisfied with the workshop overall; this increased to 96% (n = 90) in Year 2. While participant outcomes such as satisfaction are not an end unto themselves [30], participant satisfaction and agreement with the content is a useful metric for facilitators during implementation.

Year 1 levels of satisfaction with the balance of activities and lecture and length of the workshop were lower than other areas of satisfaction, indicating adjustments needed to be made. Participant feedback from open-ended survey items were used to inform the adjustments relating to balance and length. Throughout Year 2 the workshop was revised to add more activities and more time for group discussions, and the workshop length was increased. As a result, satisfaction in these areas increased from Year 1 to Year 2. Seventy-eight percent (n = 52) of Year 1 participants agreed or strongly agreed that there was an appropriate balance of activities and lecture in the workshop, while 94% (n = 88) of Year 2 participants agreed there was an appropriate balance. In Year 1 when the workshop was 3.5 hours long, 66% (n = 44) agreed or strongly agreed the length of the workshop was appropriate. In Year 2, the first three workshops delivered (Atlanta, Boulder, and Madison) were increased to 4 hours long. Based on participant feedback, the Chicago workshop was extended to 4.5 hours long, and the two Houston workshops were extended to a full 5 hours. Table 3 shows a higher average level of satisfaction with workshop length for the longer workshops. In Year 2 after the workshop was increased to 5 hours in length, 89% (n = 83) agreed or strongly agreed the length of the workshop was appropriate. Details on the changes made to the workshop content and format based on evaluation data can be found in the Lessons Learned section.

**Table 3:** Agreement with the statement: The length of this workshop was appropriate.

Workshop	Length of workshop (hours)	n	Mean
All Year 1	~less than 4	67	3.70
All Year 2	~4.5	93	4.25
Year 2: Atlanta	4	15	3.93
Year 2: Boulder	4	13	4.08
Year 2: Madison	4	19	3.89
Year 2: Chicago	4.5	14	4.50
Year 2: Houston 1	5	14	4.71
Year 2: Houston 2	5	18	4.44

Average rating on a scale of 1 = strongly disagree to 5 = strongly agree.

### Evaluation question 2: Does the developed SCOARE curriculum and workshop meet its learning objectives?

The workshop learning objectives were stated at the beginning of the workshop and reiterated throughout. The evaluation survey asked participants to rate the extent to which the learning objectives were met using a three-point scale. In Year 2, one of the learning objectives was rephrased to be more specific. Overall, the percentage of participants who rated the learning objectives as “definitely met” increased from Year 1 to Year 2 (Table 4). These increases can be attributed to adjustments in the curriculum and content such as addition of multiple examples, and addition and improvement of activities and discussions. The increases can also be attributed to the facilitators gaining experience and fine-tuning content and delivery, and evolved clarification of points.

**Table 4:** Percentage of participants who rated the learning objectives as “definitely met”.

Learning objectives	Year 1 (N = 67)		Year 2 (N = 92)	
	Freq.	Perc.	Freq.	Perc.
Understand your role and perspective as a mentor, and your trainee’s role and perspective, in SC skill development	41	62	76	83
Set expectations and create structure for your trainees in SC	45	70	79	86
Year 1: Explore and apply a variety of strategies to increase trainee engagement in SC	47	70	83	90
Year 2: Create and apply a variety of strategies to increase trainee engagement in scientific writing and speaking				
Deliver useful and appropriate feedback	39	58	73	79
Year 1: Create your own best approach	26	39	63	69
Year 2: Create, adapt and personalize your mentoring strategies to apply in your own mentoring				

### Evaluation question 3: What are participant gains in skills and knowledge from the workshop?

Participants reported positive increases in skills and knowledge post-workshop. Wilcoxon Signed Rank tests were conducted to compare the level of skills and knowledge of participants before and after the workshop.

The greatest increase in knowledge was regarding how unconscious linguistic bias influences our perception of others. Workshop participants from both Years 1 and 2 rated their level of knowledge about how linguistic biases influence perceptions of others on a scale of 0 (no knowledge) to 3 (much knowledge). The mean rating of knowledge before the workshop was 1.13 (SD = .83) and the mean level of knowledge after the workshop was 2.27 (SD = .63); this increase in knowledge is statistically significant (*Z* = 10.126, p < .001, r = .81). These results suggest that the workshop had a significant effect on the increase of participant knowledge about how linguistic biases influence perceptions of others (Table 5).

**Table 5:** Change in participant level of knowledge.

						Wilcoxon Signed Rank		
	Pre-test Mean	Std Dev	Post-test Mean	Std Dev	Pre-post Change	*Z*	*r*	Sig.
How linguistic biases influence perceptions of others*	1.13	.83	2.27	.63	+1.14	10.126	.81	p < .001
Research on the impact of scientific communication on training outcomes*	1.06	.82	2.32	.58	+1.26	10.44	.83	p < .001
Various strategies to encourage trainee engagement in scientific writing**	1.33	.67	2.53	.52	+1.20	8.423	.88	p < .001
Various strategies to encourage trainee engagement in speaking or presenting**	1.52	.69	2.54	.50	+1.02	8.004	.84	p < .001
How to avoid unproductive strategies with trainees in scientific writing**	1.12	.70	2.31	.59	+1.19	8.195	.86	p < .001
How to avoid unproductive strategies with trainees in speaking or presenting**	1.32	.81	2.30	.59	+0.98	7.394	.78	p < .001

Knowledge scale: 0 = no knowledge, 1 = low knowledge, 2 = some knowledge, 3 = high level of knowledge.

*Year 1 and Year 2 combined participants N = 158.

**Year 2 participants only N = 90 to 91.

Results suggest that the workshop had a significant effect on the increase of participant knowledge about research on the impact of SC on training outcomes. There was a statistically significant difference in the scores for level of knowledge before the workshop (M = 1.06, SD = .82) and level of knowledge after the workshop (M = 2.32, SD = .58; *Z* = 10.44, p < .001, r = .83; Table 5).

Some of the survey items about skills and knowledge were changed for Year 2 to help workshop developers determine if participants were making more gains with mentoring for scientific writing or mentoring for speaking or presenting. Tables 5 and 6 display greater changes in participant knowledge and skills about strategies related to scientific writing compared to speaking or presenting. Wilcoxon Signed Rank tests show all increases from before to after the workshop are statistically significant.

**Table 6:** Change in Year 2 participant level of skill in SC mentoring techniques.

						Wilcoxon Signed Rank		
	Pre-test Mean	Std Dev	Post-test Mean	Std Dev	Pre-Post Change	*Z*	*r*	Sig.
Providing feedback to a trainee about their scientific **writing** (n = 92)	1.85	.61	2.61	.53	+0.76	8.021	.84	p < .001
Providing feedback to a trainee about their **speaking or presenting** (n = 92)	2.18	.66	2.63	.49	+0.45	6.105	.64	p < .001
Diagnosing trainees’ needs in scientific **writing** (n = 92)	1.53	.76	2.45	.54	+0.91	7.934	.83	p < .001
Diagnosing trainees’ needs in **speaking or presenting** (n = 91)	1.93	.78	2.52	.57	+0.60	6.687	.70	p < .001
Applying new and various techniques when mentoring trainees in scientific **writing** (n = 92)	1.34	.75	2.54	.52	+1.21	8.434	.88	p < .001
Applying new and various techniques when mentoring trainees in **speaking or presenting** (n = 92)	1.54	.76	2.57	.50	+1.02	8.084	.84	p < .001
Motivating trainees to engage in scientific **writing** (n = 92)	1.45	.65	2.45	.54	+1.00	8.114	.85	p < .001
Motivating trainees to engage in **speaking or presenting** (n = 91)	1.69	.71	2.49	.52	+0.80	7.37	.77	p < .001

Skill scale: 0 = no skill, 1 = low skill, 2 = some skill, 3 = high level of skill.

Participants reported the greatest increases in skill for applying new and various techniques when mentoring trainees in scientific writing and speaking or presenting. Workshop participants from Year 2 rated their level of skill to apply new and various techniques when mentoring trainees in scientific writing on a scale of 0 (no skill) to 3 (much skill). The mean rating of skill before the workshop was 1.34 (SD = .75), and after the workshop the mean rating was 2.54 (SD = .52); this increase in skill is statistically significant (*Z* = 8.434, p < .001, r = .88). These results suggest that the workshop had a significant effect on the increase of participant skill to apply new and various techniques when mentoring trainees in scientific writing (Table 6).

On the same scale, workshop participants from Year 2 said that before the workshop their level of skill to apply new and various techniques when mentoring trainees in speaking or presenting was 1.54 (SD = .76) and 2.57 (SD = .50) after the workshop. This increase in skill is statistically significant (*Z* = 8.084, p < .001, r = .84). These results suggest that the workshop had a significant effect on the increase of participant skill to apply new and various techniques when mentoring trainees in speaking or presenting. More statistically significant increases in skill are displayed in Table 6.

### Evaluation question 4: Will participants implement what they have learned in the workshop?

The majority of Year 1 and Year 2 participants said they are very likely to use the following strategies from the workshop: create expectations and structure, increase engagement and productivity, give useful feedback, and give acknowledgement. The majority of participants also said they feel very capable of using all of the above strategies except increasing engagement and productivity. Despite feeling less capable, participants said they are still likely to use the increasing engagement strategies. Qualitative responses from participants indicate they will try their best but feel the level of engagement and productivity is also dependent on the role of the mentee.

A notable increase in feelings of capability between Year 1 and Year 2 was observed in feeling capable of giving acknowledgement. Sixty-four percent (n = 43) of Year 1 participants said they feel very capable of giving acknowledgement; in Year 2 83% (n = 77) said they feel very capable of giving acknowledgment. Qualitative responses from participants indicate increased awareness of the power of acknowledgement and how easy it is considering the impact it has on mentees.

## Discussion

Overall, the SCOARE program has been well-received by both faculty and staff mentors. Through the SCOARE program, we have already trained over 150 faculty and staff to address their trainees’ SC skill development. This mentoring intervention, which targets the “Mentoring in SC” construct of our SC-adapted SCCT model, is an important step towards increasing trainees’ research career persistence. Positive results from the workshop evaluations described in this report, with increased faculty confidence, skills, and knowledge to implement SC mentoring, as well as our preliminary research study data suggest that mentor gains from the workshop are positively impacting trainees and that SC skill development mentoring workshops like the SCOARE workshop can have a measurable impact on trainee research career intention. As we continue to gather and analyze our research study data, we hope to shed light on the details of this impact.

The lessons we learned from our workshop evaluation data fall into three main categories: balancing content covered and workshop length, setting and managing audience expectations, and addressing audience concerns.

### Balancing content covered and workshop length

Over the first two years of workshop delivery, we honed the content and length of the workshop in response to mentor feedback, working to balance the time spent on the didactic workshop sections with activities, group discussions, and individual reflection time. While quantitative ratings of workshop length from Year 1 participants were acceptable, workshop delivery felt rushed, and there were a number of qualitative comments noting a lack of time to digest material and interact with other participants. To this end, in Year 2 we both extended the total workshop length while also reducing the amount of material covered or finding more efficient ways to share while still achieving the objectives of the workshop. We landed on a 5-hour workshop as the ideal length; this was short enough that participants did not feel like it took up their entire day and long enough that there was sufficient time for participants to digest the material, have discussions, and learn from other attendees’ experiences without feeling rushed. No decrease in workshop registration was observed after the extension of length.

In response to feedback on the evaluations, we also reduced the amount of time spent on the research basis for SC skill mentoring. While important, details about the research often sidetracked the workshop into extended conversations, cutting down on the time available to cover practical mentoring strategies. Acknowledging the research basis for the workshop without going into extensive detail provided credibility for the contents of the workshop while allowing mentors maximum workshop time to learn about new strategies they could take and put into practice immediately. Importantly, removal of some of these research details from the presentation did not diminish overall workshop satisfaction.

By increasing workshop length and reducing or streamlining workshop content, we were able to find a balance between didactic delivery of material, group discussion and processing of material, and individual reflection which translated into greater participant satisfaction.

### Setting and managing audience expectations

The SCOARE workshop focuses on strategies to mentor all three modes of SC—speaking, presenting, and writing—for all trainees, but we found that attendees often signed up for the workshop looking for a subset of specific strategies. These ranged from strategies specific to editing trainees’ writing to strategies for non-native English speakers to cure-alls to get trainees immediately more engaged in and better at SC. Our strategy to deal with this was multifaceted. First, in the workshop introduction, we emphasized the focus on all modes of communication and on incremental changes that build from strong mentoring practices (no quick fixes). This included a short table discussion to have participants surface the top SC challenges they face with trainees. We came back to the responses from this exercise at the end of the workshop to make sure everyone’s challenges had been addressed. We also emphasized that the goal of the workshop was to learn *mentoring* skills, not *corrective* skills. Second, we took the audience pulse mid-way through the workshop with an activity in which participants wrote on a Post-it note what they were most surprised by, most unsettled by, and still puzzled by. These Post-it notes were collected on flipchart pages on the wall before the lunch break. This allowed us to surface any major audience concerns from the first half of the workshop, so we could address them in the second half. Third, we added in an extended exercise in Year 2 addressing how to help a trainee distinguish key concepts and elements of a research report. The exercise could also be used to analyze an existing piece of writing. It gave mentors who were most concerned with writing a relevant, concrete takeaway that they could immediately use with their trainees.

Lastly, we reframed our module on productivity and engagement to emphasize that engagement is built over time with the use of a combination of all the strategies covered in the workshop. This helped mentors understand that if they can get their trainees incrementally more involved in all modes of SC early on and continue throughout their training (and themselves refrain from overwriting the trainee’s work), improvement will come with time. Adding or changing existing exercises within the workshop to focus on collecting audience concerns and addressing them allowed us to both set and manage the expectations of our workshop attendees. Audience hopes for “quick fixes” were negotiated with clear communication about the incremental nature of change in trainee SC skill and the note that a little extra SC mentoring can go a long way.

### Addressing audience concerns

Participant concerns varied from audience to audience, depending on the makeup of each group (faculty vs. staff, years of experience mentoring, discipline, etc.). Our main strategy to address these concerns was to add in more group discussion and personal reflection time to increase both the engagement and connectivity of the participants and to ensure we had time to address all audience concerns. Two of the most common concerns were a sense of pressure to take dramatic, complex actions to improve their SC mentoring and worries about the viability of enacting the strategies they were learning about outside of the workshop environment.

To address the former, we highlighted small but impactful actions mentors could take, especially strategies to acknowledge trainee effort. We also emphasized that trainee engagement is built over time by simply getting the trainee producing more SC products, whether they are ‘formal’ products (e.g., a manuscript for submission) or ‘informal’ (e.g., a summary of new literature at a lab meeting). To address the latter, we streamlined the mentor ‘Brainstorming Sheet’ to help mentors concretely plan out which strategies they wanted to try, the situations they want to try them in, and what additional resources they might need to take this action. We gave participants more frequent reminders to make notes on this sheet throughout the workshop and dedicated the last 15–20 minutes of each workshop as “planning time” for mentors to formalize their strategies for moving forward. To ensure they completed their plans, as a final activity, participants paired up with a colleague that they had not yet spoken with and ‘presented’ their plans to each other. Participants were highly engaged in this activity.

Even with these changes, the evaluations showed that participants still struggle with confidence and feelings of capability in using the strategies related to increasing engagement and productivity. This is not an uncommon participant reaction to learning new material and realizing how much there is to master it. To support participants, we direct them to the extensive resources on our website (including complete instructions for all activities presented), which we continue to update with additional material on these subjects. Participants are encouraged to contact the team for guidance at any time.

### Limitations

The main limitation of our report is that because the data discussed are evaluation data rather than research data, the findings are not fully generalizable but are rather intended specifically to improve the implementation of the SCOARE workshop. Indeed, the items on our evaluation surveys were tailed to address the SCOARE workshop’s specific learning objectives. However, we feel that the lessons learned from our iterative evaluation process are applicable and of use and interest for other mentor training workshops.

Another limitation concerns our recruitment population. First, as we were using site-specific recruitment, we were limited in what regions of the country our workshop participants were from, and we did not have diversity of participant region in any one workshop. We also gave priority registration to faculty who could participate in the research study, so our population is skewed for faculty who were currently mentoring graduate students and/or postdoctoral fellows. Lastly, as an entirely optional training, we may not have reached all of the research mentors who might benefit from this training. For optional opportunities like this one, the people who sign up tend to be those who are already heavily involved in mentoring and are seeking additional tools and strategies to add to a toolbox that is already very actively used with trainees. In spite of these recruitment population limitations, we saw a distribution of both prior experience and growth in our workshop attendees.

### The future of SCOARE

We will continue to deliver SCOARE workshops and collect research study data at our national partner sites, as well as on request at other institutions or conferences, through 2022. In addition to SCOARE mentor workshops, we have begun to deliver SCOARE facilitator training workshops (“Train-the-Trainer”), which will also run through 2022. Train-the-Trainer materials and curriculum were designed taking the workshop evaluation recommendations into account, and an evaluation of the Train-the-Trainer is being conducted as well. With the goal of training 10–15 new facilitators each year, we hope to increase the impact of the SCOARE program by scaling up its reach and making the workshop more widely available to all mentors.

After the conclusion of our research study, all workshop materials, including our resource website for mentors and trainees and the Train-the-Trainer materials from our facilitator training workshop, will be made open access and available to the public. In addition, a spin-off workshop with similar content is under development for a research trainee audience. Lastly, analysis of data from the associated dyadic research study of mentors and trainees is underway and will continue until the last wave of surveys, six months after the final workshop. Comparisons of measures pre- and post-workshop will be conducted to identify changes in mentor behaviors and expectations as well as changes in mentee perceptions of mentor behaviors and expectations and changes in mentee research career intention.

## Supporting information

S1 File Post SCOARE workshop evaluation survey. (PDF)

S1 Dataset Full SCOARE workshop evaluation survey data. (XLSX)
