# Supplementary material for: Translating mentoring interventions research into practice: Evaluation of an evidence-based workshop for research mentors on developing trainees’ scientific communication skills
Source: PLoS One. 2022 Feb 2;17(2):e0262418. doi: 10.1371/journal.pone.0262418 (PMC8809591; doi:10.1371/journal.pone.0262418)
Supplement: S1 File — (PDF) [file pone.0262418.s001.pdf]

Evaluation of the SCOARE Workshop

Scientific Communication Advances Research Excellence

[location], [city], [state] – [date]

Please indicate your title/role:

- ❑ Professor **circle:** full associate assistant
- ❑ Staff **describe:**________________________
- ❑ Postdoctoral researcher
- ❑ Other:____

Approximately how many trainees do you currently mentor? __________________________

How long have you been mentoring trainees? _______________________________________

In what field or discipline do you primarily mentor trainees? __________________________

Are you a SCOARE study participant? **YES** ❑ **NO** ❑

Please indicate your level of agreement with the following statements:

	Strongly Disagree	Disagree	Neither Disagree nor Agree	Agree	Strongly Agree
I learned a lot from the workshop.	❑	❑	❑	❑	❑
The workshop increased my understanding of mentoring trainees in scientific communication.	❑	❑	❑	❑	❑
There was an appropriate balance of activities and lecture in this workshop.	❑	❑	❑	❑	❑
The length of this workshop was appropriate.	❑	❑	❑	❑	❑
The workshop facilitators were effective.	❑	❑	❑	❑	❑
I would recommend this workshop to a colleague.	❑	❑	❑	❑	❑
I am satisfied with this workshop overall.	❑	❑	❑	❑	❑

Please explain any of your responses to the above:

Please indicate how well each of the following workshop objectives was met:

	This was definitely met	This was somewhat met	This was not at all met
Understand **your role and perspective** as a mentor, and the **trainee's role and perspective**, in scientific communication (SC) skill development	❑	❑	❑
Set **expectations and create structure** for your trainees in scientific writing and speaking	❑	❑	❑
Create and apply a variety of strategies to **increase trainee engagement** in scientific writing and speaking	❑	❑	❑
Deliver useful and appropriate **feedback**	❑	❑	❑
Create, adapt and personalize mentoring strategies to apply in your own mentoring	❑	❑	❑

Please indicate your level of <u>skill</u> in the following areas *BEFORE* attending this workshop, and *NOW*:

	Skill BEFORE				Skill NOW			
	No	Low	Some	High	No	Low	Some	High
**Providing feedback to a trainee about their…**								
Scientific writing	❑	❑	❑	❑	❑	❑	❑	❑
Speaking or presenting	❑	❑	❑	❑	❑	❑	❑	❑
**Diagnosing trainees' needs in…**								
Scientific writing	❑	❑	❑	❑	❑	❑	❑	❑
Speaking or presenting	❑	❑	❑	❑	❑	❑	❑	❑
**Applying new and various techniques when mentoring trainees…**								
Scientific writing	❑	❑	❑	❑	❑	❑	❑	❑
Speaking or presenting	❑	❑	❑	❑	❑	❑	❑	❑
**Motivating trainees to engage in…**								
Scientific writing	❑	❑	❑	❑	❑	❑	❑	❑
Speaking or presenting	❑	❑	❑	❑	❑	❑	❑	❑

Please indicate your level of <u>knowledge</u> in the following areas *BEFORE* attending this workshop, and *NOW*:

	Knowledge BEFORE				Knowledge NOW			
	No	Low	Some	High	No	Low	Some	High
**Research about the impact of scientific communication on training outcomes**	❑	❑	❑	❑	❑	❑	❑	❑
**How linguistic biases influence our perception of others**	❑	❑	❑	❑	❑	❑	❑	❑
**Various strategies to encourage trainee engagement in…**								
Scientific writing	❑	❑	❑	❑	❑	❑	❑	❑
Speaking or presenting	❑	❑	❑	❑	❑	❑	❑	❑
**How to avoid unproductive strategies with trainees in…**								
Scientific writing	❑	❑	❑	❑	❑	❑	❑	❑
Speaking or presenting	❑	❑	❑	❑	❑	❑	❑	❑

What other skills or knowledge did you gain from participating in this workshop?

What was the most important thing you learned from participating in this workshop?

What did you expect or hope to learn, but did not?

How likely are you to use any of the following strategies with a trainee to improve their scientific communication?

	Not at all Likely	Somewhat Likely	Very Likely
Create expectations and structure	❑	❑	❑
Increase engagement and productivity	❑	❑	❑
Give useful feedback	❑	❑	❑
Give acknowledgement	❑	❑	❑

How capable do you feel to use any of the following strategies with a trainee to improve their scientific communication?

	Not at all Capable	Somewhat Capable	Very Capable
Create expectations and structure	❑	❑	❑
Increase engagement and productivity	❑	❑	❑
Give useful feedback	❑	❑	❑
Give acknowledgement	❑	❑	❑

Which of the above strategies will be most difficult to implement for you? Please explain.

Please comment on the logistics, including time of workshop, location, parking, refreshments, breaks, registration process, communication with planners:

Please provide additional feedback about this workshop, including suggestions for improvement.

Thank you! Please return this evaluation immediately to your workshop presenter or evaluator.

Christine Bell
cfabian2@wisc.edu

Wisconsin Center for Education Research
1025 W. Johnson Street - Suite #552
Madison, WI 53706
